# Study on Kiln-Transformation Mechanism of 3D-Printed Body of Hejin Gray Pottery

**DOI:** 10.3390/ma19143063

**Published:** 2026-07-16

**Authors:** Shuai Liu, Wenjie Hao, Guolong Gao, Yu Liu, Hanjie Guo, Yongsheng Zhou, Jiafeng Lv, Yalin Liu

**Affiliations:** 1 School of New Materials and Future Technology, Beijing Technology and Business University, Beijing 100048, China; 20210720@btbu.edu.cn; 2 School of Computer and Artificial Intelligence, Beijing Technology and Business University, Beijing 100048, China; 17732726224@163.com (W.H.);; 3 School of Metallurgical and Ecological Engineering, University of Science and Technology Beijing, Beijing 100083, China; 4 Industrial Informatization and Science and Technology Bureau of Hejin Municipal People’s Government, Hejin 043300, China; 5 Hejin Longmen Lvshi Brick and Glass Carving Co., Ltd., Hejin 043300, China; longmenlvshi@163.com

**Keywords:** Hejin gray pottery, direct ink writing, kiln transformation, thermodynamic analysis, phase evolution, FeAl_2_O_4_ spinel

## Abstract

The firing of traditional gray pottery relies on complex physicochemical reactions governing its color, dimensional accuracy, and structural stability. Unclear kiln-transformation mechanisms restrict standardized and digital production of this Chinese intangible cultural heritage. Herein, direct ink writing (DIW) was used to fabricate Hejin gray pottery green bodies from local ternary raw materials. Thermodynamic calculations, TG–DTG/DSC, XRD, XRF, and atmosphere-controlled firing tests were combined to reveal coupled phase evolution and reduction color-forming mechanisms during sintering. Two interrelated kiln-transformation processes were identified. First, sequential mineral reconstruction occurs at four critical temperatures: free water loss at 119.8 °C, two-stage dehydroxylation of hydrous silicates at 270.5 °C and 767.9 °C, and CaCO_3_ decomposition at 547.9 °C. Uneven shrinkage and gas release at these temperatures induce cracking, blistering, and deformation of printed bodies. Micron-sized CaCO_3_ (equivalent radius ≈ 1.31 μm) exhibits high surface energy and significantly reduces its decomposition temperature, consistent with experimental observations. Second, reducing atmospheres trigger competitive phase formation. Distinct from the conventional Fe_2_O_3_ → Fe_3_O_4_ → FeO reduction pathway, Fe oxides preferentially react with abundant Al_2_O_3_ to form thermodynamically stable FeAl_2_O_4_ spinel, yielding uniform celadon-gray tones. The final color is nearly independent of 20–90 vol% CO, and air-isolated cooling below 600 °C is mandatory to prevent secondary oxidation and reddening. This work establishes a thermodynamic framework for DIW-printed Hejin gray pottery kiln transformation, clarifies microscale defect and color-evolution mechanisms, and offers theoretical guidance for atmosphere-controlled firing and digital mass production of heritage ceramics.

## 1. Introduction

Ceramics have long served as important carriers of human civilization because of their unique combination of cultural significance, structural durability, and architectural functionality [1,2]. Among traditional Chinese ceramics, Hejin gray pottery is a representative architectural material manufactured from locally sourced purple sand, steel mud, and clay under a reducing firing atmosphere, which produces its characteristic bluish-gray appearance. Owing to its dense microstructure, excellent weather resistance, and ornamental value, it has been extensively used in brick carvings, roof ornaments, and antique architectural decorations. The firing technique of Hejin gray pottery was inscribed on the National Intangible Cultural Heritage List of China in 2008, and Hejin was officially recognized as the “Hometown of Grey Pottery Glaze Culture in China” in 2020. Despite its important cultural and engineering value, the production of Hejin gray pottery still relies heavily on empirical craftsmanship, making it difficult to achieve standardized manufacturing and quality consistency.

Modernization of Hejin gray pottery is currently hindered by three major technical challenges. First, the absence of standardized raw material characterization and composition design results in poor batch-to-batch consistency. Second, conventional hand-forming methods exhibit low manufacturing efficiency and are unsuitable for fabricating complex geometries with high dimensional accuracy [3,4,5]. More importantly, the physicochemical mechanisms governing kiln transformation remain unclear, leading to frequent firing defects such as cracking, deformation, blistering, and color inconsistencies [6]. These limitations substantially restrict process optimization, product reliability, and large-scale industrial production.

Recent advances in additive manufacturing have provided new opportunities for the digital preservation and intelligent manufacturing of traditional ceramics [5]. Among various ceramic additive manufacturing technologies, direct ink writing (DIW) has become one of the most widely adopted techniques because of its low equipment cost, ambient-temperature processing, and excellent compatibility with clay-based materials [3]. Previous studies have demonstrated that DIW can successfully fabricate complex ceramic components based on clay, alumina, zirconia, and silicon carbide by optimizing slurry rheology, solid loading, and printing parameters [4,7,8,9]. Meanwhile, thermal characterization techniques such as TG–DSC and X-ray diffraction (XRD) have been extensively employed to investigate phase evolution during ceramic firing [10,11,12], whereas thermodynamic approaches based on the Ellingham diagram have been widely applied to describe the reduction behavior of iron oxides under reducing atmospheres [13]. More recently, atmosphere-controlled firing and phase evolution of additively manufactured ceramics have attracted increasing attention because the firing atmosphere critically influences densification, microstructure, and functional performance [10].

However, existing studies mainly focus on high-purity engineering ceramics or conventional clay systems, while the firing behavior of iron-rich multicomponent traditional ceramics remains poorly understood [3,5]. In particular, the raw materials of Hejin gray pottery contain unusually high concentrations of Fe_2_O_3_, Al_2_O_3_, and CaO, giving rise to complex interactions among mineral decomposition, iron oxide reduction, phase competition, and color development during firing [14]. These coupled physicochemical processes differ fundamentally from those in conventional ceramic systems and cannot be adequately explained by the traditional stepwise reduction pathway of Fe_2_O_3_ → Fe_3_O_4_ → FeO alone [15]. Furthermore, DIW-fabricated ceramic bodies possess unique layered architectures, pore distributions, and sintering characteristics, suggesting that their kiln-transformation behavior may differ significantly from that of conventionally formed ceramics [5]. Consequently, the intrinsic relationship among thermal evolution, phase transformation, reduction atmosphere, and final coloration has not yet been established, which remains a critical scientific obstacle to the digital manufacturing of Hejin gray pottery.

To address these challenges, this study employs locally sourced purple sand, steel mud, and clay to fabricate Hejin gray pottery bodies using DIW technology. Thermodynamic calculations are integrated with TG–DTG/DSC, X-ray diffraction (XRD), X-ray fluorescence (XRF), and atmosphere-controlled firing experiments to systematically investigate the coupled evolution of mineral phases and iron-bearing species throughout the firing process. The main scientific contributions of this work are threefold. First, the sequential thermal evolution and phase transformation of DIW-printed Hejin gray pottery are clarified over the entire firing process. Second, the thermodynamic competition between iron oxide reduction and Fe–Al spinel formation is established, demonstrating that the formation of thermodynamically stable FeAl_2_O_4_, rather than the conventional Fe_3_O_4_/FeO pathway, dominates the stabilization of the characteristic bluish-gray color. Third, the intrinsic relationship between firing temperature, reducing atmosphere, phase evolution, and color development is elucidated, providing a thermodynamic framework for atmosphere-controlled firing and a scientific basis for the digital manufacturing, quality control, and industrial modernization of traditional iron-rich ceramic materials.

## 2. Experimental Materials, Equipment, and Methods

### 2.1. Experimental Materials

All experimental materials were taken from the traditional gray pottery raw material production area in Hejin City, Shanxi Province, including three natural mineral raw materials: purple sand, clay, and steel mud. Their main chemical compositions are shown in Table 1.

**Figure 1:**
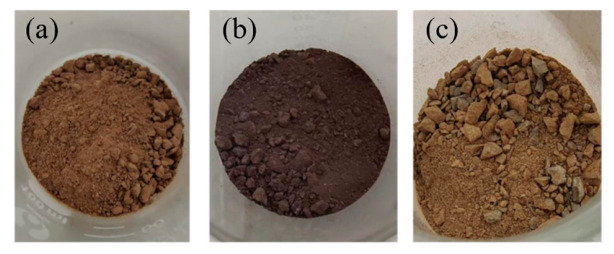
Three raw materials of Hejin gray pottery: (**a**) clay; (**b**) purple sand; (**c**) steel mud.

**Figure 2:**
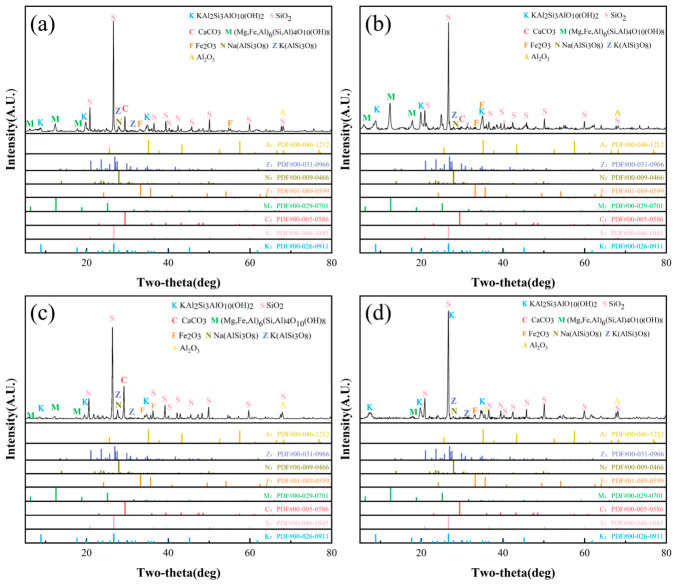
XRD patterns of the mixed material and three raw materials: (**a**) 1:1:1 mixture; (**b**) steel mud; (**c**) clay; (**d**) purple sand. All characteristic diffraction peaks of the main mineral components are clearly marked for phase discrimination.

The original colors of the three raw materials are shown in Figure 1. Among them, purple sand is rich in SiO_2_ and Fe_2_O_3_. Fe_2_O_3_ is the key to color during firing: Fe_2_O_3_ is red, gray when reduced to Fe_3_O_4_, and black when further reduced to FeO. The high content of Al_2_O_3_ in steel paste can affect the degree of reduction of Fe_2_O_3_, thereby indirectly regulating the color of the product. Clay is rich in CaO, has strong adhesion, and contains CaCO_3_. The decomposition process of its crystal structure will affect the firing process and is a key factor in controlling the cracking of the green body [10]. Some minerals containing crystalline water in the raw materials can also affect the firing process. To clarify the phase composition and crystal structure of the raw materials and mixtures, XRD analysis was performed on three types of raw materials and mixed samples, and the results are shown in Figure 2. The phase identification of sintered samples and raw mineral mixtures was realized by matching diffraction peaks with the standard ICDD PDF-4 mineral database via Jade 9 software. All characteristic diffraction peaks corresponding to crystalline mineral phases were labeled on the collected XRD patterns for intuitive phase comparison.

The three raw materials are mixed in a traditional process with a quality ratio of 1:1:1, serving as the base material.

As shown in Figure 2, purple sand, clay, steel mud, and mixtures all contain high levels of SiO_2_, with the highest diffraction peak intensity. The peak intensity of (Mg,Fe,Al)_6_(Si,Al)_4_O_10_(OH)_8_ in steel mud is prominent, which is a composite compound containing crystalline water. When fired at the temperature of crystalline water decomposition, it leads to rapid volume expansion due to the release of crystalline water, resulting in cracks. The decomposition temperature of crystalline water needs to be studied. The CaCO_3_ present in loam releases CO_2_ at its decomposition temperature during firing, which is also a primary cause of cracks. In purple sand, the highest intensity of Fe_2_O_3_ is the key factor for color change in the kiln and the theoretical basis for the formation of gray pottery.

To further clarify the phase identification, the main diffraction peaks in Figure 2 were indexed according to the ICDD PDF-4 database and compared with reported clay-based ceramic systems. The intense reflection at 2θ ≈ 26.6° was assigned to quartz (101), while the basal reflections near 12.3° and 8.8°/17.8° were attributed to kaolinite and illite, respectively. Hematite was identified by its characteristic reflections near 33.1° (104) and 35.6° (110), whereas calcite, where present, was recognized from the diagnostic peak at 29.3° (104). A similar mineral assemblage of quartz, kaolinite, illite, calcite, and hematite has been reported for Chinese red clays used in ceramic and construction materials [16], supporting the reliability of the present phase assignment.

### 2.2. Raw Material Processing and Experimental Protocol

**Figure 3:**
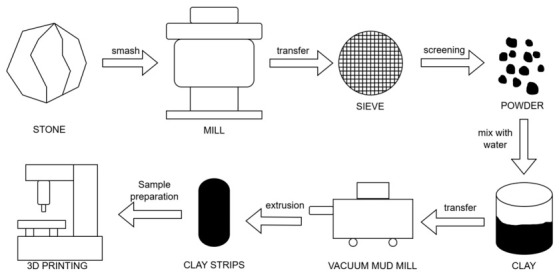
Raw material processing and 3D printing process.

The raw material processing and experimental process are shown in Figure 3.

The pre-treatment process for raw materials includes the following: (1) crushing → (2) ball milling → (3) sieving through a 200-mesh sieve → (4) mixing with water → (5) vacuum refining → (6) aging. The final moisture content of the blank is controlled at 24–27% to meet the rheological requirements of extrusion 3D printing.

### 2.3. DIW Forming Process and Structural Characteristics of Printed Green Bodies

An extrusion 3D printer (Smart PN-2030, Xiamen Smart Industrial Design Co., Ltd., Xiamen, China) was employed, with a forming size range of ≤200 mm × 200 mm × 300 mm.

The fixed printing parameters were as follows: a printing speed of 40 mm/s; layer thickness of 0.7 mm; filling rate of 70%. The sample was printed as a 10 mm × 10 mm × 10 mm sample, and the printed body was dried naturally at room temperature for 3–4 days to ensure sufficient removal of free water and to avoid cracking during firing. After drying, firing was carried out in electric kilns with different atmospheres to study the kiln-transformation mechanism of gray pottery under different firing conditions, providing a theoretical basis for the optimal firing process.

**Figure 4:**
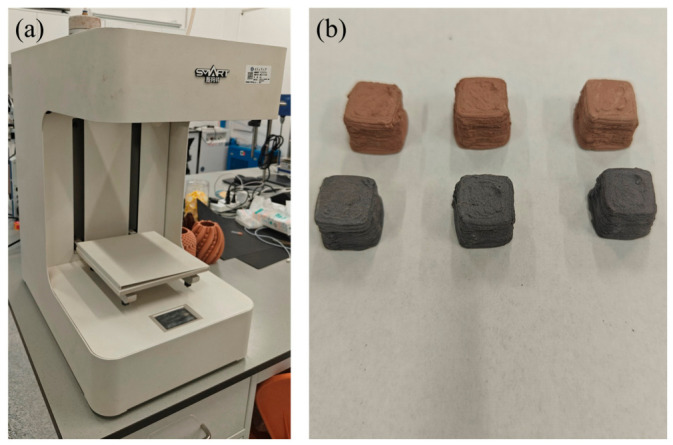
(**a**) Extrusion-based direct ink writing (DIW) printer (Model Smart PN-2030) used for preparing the ceramic green bodies; (**b**) photographs of the 3D-printed cubic samples (10 × 10 × 10 mm): the upper three samples are in the air-dried green state, and the lower three samples are the corresponding specimens after firing at 900 °C under a 50 vol% CO atmosphere.

The DIW molding apparatus and dried cubic specimens used in the present study are displayed in Figure 4.

Unlike traditional forming methods (slip casting, hand molding), direct ink writing (DIW) builds parts layer-by-layer, imparting unique structural features that critically influence subsequent kiln transformation [3].

At the mesoscopic scale, the printed body exhibits a lamellar architecture with interlayer interfaces and interfilament voids. This anisotropic configuration leads to non-uniform shrinkage during drying and early heating, with stress concentrating along weakly bonded layer boundaries—a primary source of differential deformation not typically observed in isotropic molded bodies.

At the microscopic scale, the high water content (24–27 wt%) required for extrudability leaves residual micropores within filaments and at interfaces. During heating, rapid vaporization of this moisture (corresponding to the 119.8 °C endotherm in Section 3.1) can build internal pressure, yet the layered “channel” structure may hinder smooth gas escape, promoting blistering and micro-cracks.

More critically, this architecture exacerbates defects at higher temperatures where gas-releasing reactions occur: dehydroxylation (270.5 °C, 767.9 °C) and CaCO_3_ decomposition (547.9 °C) generate H_2_O and CO_2_ that must travel along long pathways through weak interlayer interfaces. When gas production outpaces permeation—especially at 70% infill—pressure builds preferentially along these planes, inducing delamination and interlayer cracking [10]. Such defect modes are characteristic of DIW parts but rare in conventional bodies, which offer more isotropic gas permeation.

Additionally, extrusion-induced preferential alignment of plate-like clay minerals along the printing direction may produce anisotropic thermal expansion and sintering shrinkage, potentially affecting dimensional accuracy and phase distribution (including FeAl_2_O_4_ spinel formation, which is discussed in Section 4).

These DIW-specific features—layered interfaces, constrained gas pathways, and particle orientation—provide the structural foundation for understanding the defect mechanisms at the four critical temperature nodes identified in Section 3 and guide the design of optimized firing schedules for printed Hejin gray pottery.

### 2.4. Raw Material Characterization and Firing Conditions

The phase composition of the raw materials was analyzed by X-ray diffraction (XRD) (Bruker AXS, Karlsruhe, Germany) using a Cu-target X-ray tube (Kα radiation, λ = 1.5406 Å) operated at 40 kV and 40 mA. Data were collected over a 2θ range of 5–80° with a step size of 0.02° and a scanning speed of 2°/min. Phase identification was performed by matching the observed diffraction peaks with the ICDD PDF-4 database using Jade 9 software. The elemental oxide composition of the raw materials was determined by X-ray fluorescence (XRF) spectroscopy(Bruker AXS, Karlsruhe, Germany).

Thermal decomposition and mass loss behaviors of the raw materials were characterized by synchronous thermal analysis (STA) (Netzsch, Selb, Germany) under an argon atmosphere at a heating rate of 10 °C/min from room temperature to 1000 °C, with an alumina crucible and a sample mass of approximately 10–15 mg.

After drying, the specimens were fired in atmosphere-controlled electric kilns (Xiamen Smart Industrial Design Co., Ltd., Xiamen, China) with an adjustable CO volume concentration ranging from 0 vol% to 90 vol% and a temperature accuracy of ±5 °C. The firing temperatures, holding times, and atmosphere conditions were determined based on the thermal analysis and variable-temperature XRD results.

## 3. Simulation of Molecular Structure Changes and Characterization of Raw Materials During the Firing Process of Gray Pottery

In this study, the evolution of molecular structures of three raw materials—steel mud, clay, and purple sand—as well as 3D-printed samples of their 1:1:1 mixture, was systematically investigated over the firing temperature range from room temperature to 1000 °C using combined TG–DTG, DSC, XRD, and XRF analyses [17,18]. For structural characterization, a semi-quantitative approach based on XRD peak shifts, relative diffraction intensities, and full width at half maximum (FWHM) was adopted, a method widely accepted in clay mineralogy and traditional ceramic research [16,19]. Furthermore, by integrating the thermal evolution curves from TG–DTG with thermodynamic calculations of Gibbs free energy, the coupled transformation mechanism of the DIW-printed Hejin gray pottery during kiln firing is comprehensively elucidated. First, we studied the evolution law of molecular structure during the firing process and then characterized the molecular structure at different stages.

### 3.1. Evolution and Characterization of Molecular Structure in Raw Materials During Firing

**Figure 5:**
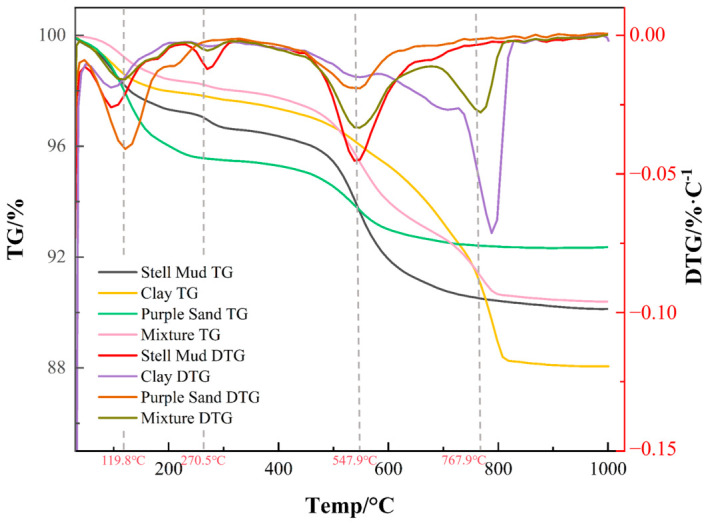
TG and DTG curves of the three raw materials and their 1:1:1 mixture from RT to 1000 °C in an Ar atmosphere. Vertical dashed lines mark the four critical temperature nodes (119.8 °C, 270.5 °C, 547.9 °C, and 767.9 °C) corresponding to free water evaporation, dehydroxylation, CaCO_3_ decomposition, and complete dehydroxylation, respectively.

**Figure 6:**
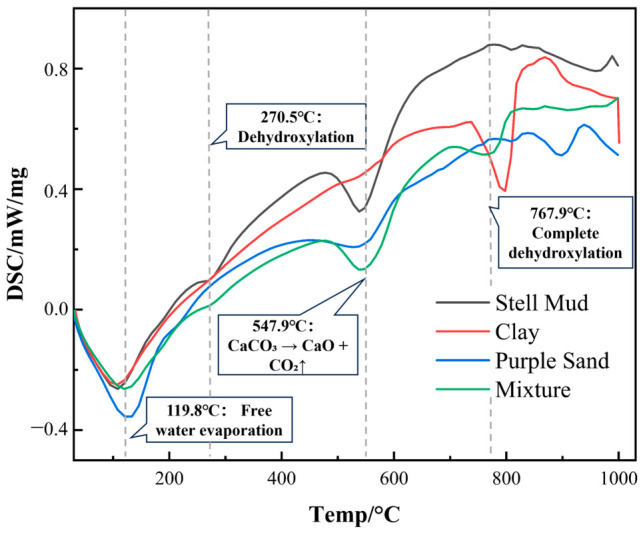
DSC curves of individual raw materials and the 1:1:1 mixture (heating rate: 10 °C/min, Ar atmosphere). Downward peaks indicate endothermic reactions. The four characteristic endothermic peaks of the mixture, located at 119.8 °C, 270.5 °C, 547.9 °C, and 767.9 °C, correspond to free water evaporation, dehydroxylation of hydrous silicates, CaCO_3_ decomposition, and complete dehydroxylation to form metakaolin, respectively.

Place the sample in an argon environment and heat it up from room temperature to 1000 °C in a thermal analyzer at a heating rate of 10 °C/min. Obtain the TG/DTG and DSC curves of 3D-printed samples of three raw materials (steel mud, clay, purple sand) and mixtures, as shown in Figure 5 and Figure 6. The horizontal axis in Figure 5 represents the temperature (Temp), ranging from 25 to 1000 °C. The left vertical axis represents TG (thermal weight percentage, the proportion of remaining mass to initial mass,%), with lower values indicating more weight loss. The vertical axis on the right is DTG (Thermogravimetric Differential, Weight Loss Rate, % °C^−1^), and the deeper the valley value, the faster the weight loss reaction rate at that temperature.

Figure 5 presents the thermogravimetric (TG) curves of the three raw materials and their 1:1:1 mixture. The clay exhibits the highest total weight loss, with a residual mass of only ~88% at 1000 °C, corresponding to a total weight loss of ~12%. This is primarily attributed to the combined decomposition of CaCO_3_ (releasing CO_2_) and dehydroxylation of hydrous silicates. The steel-making sludge shows an intermediate weight loss (~10%), mainly due to the decomposition of layered silicate phases, while the purple clay shows the lowest weight loss (~7.8%), consistent with its relatively low content of volatile-bearing phases.

The TG curve of the ternary mixture is approximately the weighted average of its three components, indicating that no significant synergistic interactions occur during heating below 850 °C. This linear additivity validates the representativeness of the mixture for subsequent mechanistic studies.

Compared with conventional clay-based ceramics reported in the literature, which typically show total weight losses of 5–8% over the same temperature range, the Hejin gray pottery raw materials exhibit a notably higher mass loss. Fotsop et al. [20] reported a total mass loss of ~12.5% for Cameroonian kaolin, attributed to dehydration and dehydroxylation, which is comparable to the ~12% loss observed for the clay component in this study. González-Miranda et al. [21] further demonstrated that in sericite–kaolinite clays, the dehydroxylation peaks of kaolinite and sericite overlap, resulting in a broadened endothermic event with the main mass loss occurring in this overlapping region—consistent with the complex weight loss profile observed for the clay component. This compositional feature necessitates a more carefully designed heating schedule to mitigate the risk of cracking or bloating during firing.

From the DTG curve in Figure 5 and the DSC curve in Figure 6, it can be observed that the raw material exhibits four weight loss peaks at different temperatures during the heating process. The following analysis will be conducted on each peak.

(1) At <200 °C: The characteristic temperature of the mixture appears at 119.8 °C (0.0159%/°C, where water evaporates from the material), indicating a physical water removal stage. The weight loss of purple sand is more significant, with a mass loss rate of 7.64%. The DTG peak is 120.9 °C (0.041%/°C), and the weight loss of clay and steel mud is extremely weak in this range, indicating that there is less physical water contained in it. The theoretical evaporation temperature of physical water is 100 °C, which is a phase transition from liquid to gas. At this time, the temperature of the system should remain unchanged. However, due to the artificially set heating rate of 10 degrees per minute, the displayed temperature is 19.8 °C higher than the theoretical temperature.

The predominant weight loss of purple sand can be attributed to its high Fe_2_O_3_ content (44.2%) and associated high specific surface area, which facilitates the adsorption of substantial interparticle water. This is consistent with the findings of Mostafa et al. [18], who reported that free water content correlates positively with specific surface area rather than bulk composition. The slight enhancement of all mineral diffraction peaks at 200 °C originates from the removal of free interparticle water, which makes the stacking of mineral particles tighter without any destruction of crystal lattices. This explains why only slight shrinkage rather than cracking occurs for DIW-printed green bodies below 200 °C, a behavior also observed by Bhandari et al. [10] in DIW-fabricated alumina ceramics during low-temperature drying.

(2) At 200~400 °C: The characteristic temperature of the mixture appears at 270.5 °C (0.005%/°C), which is the stage of crystal water and hydroxyl removal. The weight loss of clay and purple sand is more obvious, and the interlayer bound water and structural hydroxyl groups are gradually lost; the weight loss of steel mud in this range is extremely weak, indicating that there is basically no crystal water in it.

This dehydroxylation temperature is notably lower than the typical 450–600 °C range for pure kaolinite reported by Fan et al. [17] and Sánchez-Soto et al. [19]. The reduction is attributed to Fe^3+^ and Al^3+^ substitutions in octahedral sites weakening the M–O–H bond strength. The d-spacing contraction (~3–5%) has been quantitatively documented by Gu et al. [16] in Chinese red clay upon dehydroxylation. The inherent layered pore structure of 3D-printed blanks will produce uneven volume shrinkage in this temperature interval, easily triggering tiny surface cracks during firing if the heating rate is not carefully controlled.

(3) At 400~850 °C: The weight loss peak of the 1:1:1 mixture is significant, resulting from the combined decomposition reactions of the three raw materials. The characteristic temperatures are 547.9 °C (0.0332%/°C) and 767.9 °C (0.0277%/°C). Before 600 °C, the weight loss rate of purple sand was the highest due to carbonate decomposition (peak at 548.1 °C, 0.019%/°C); afterwards, clay dominates the weight loss due to kaolinite dehydroxylation, while calcium hydroxide and trace calcium silicate in steel mud slowly decompose. The CaCO_3_ decomposition temperature of 547.9 °C is remarkably lower than the 700–900 °C range reported for calcite in ceramic systems [22,23], corroborated by Rodriguez-Navarro et al. [24], who observed nanocrystalline CaCO_3_ decomposition at ~735 °C. Combined with the particle surface energy calculation in Section 3.2, micron-sized CaCO_3_ (equivalent radius 1.31 μm) exhibits high surface activity that drastically lowers its decomposition temperature. The instantaneous CO_2_ release upon CaCO_3_ breakdown, combined with the limited gas permeability of the layered DIW structure, is the primary origin of blister and delamination defects—consistent with Ondruška et al. [25], who linked CO_2_ retention to closed pore formation.

(4) At >850 °C: Thermal stability and sintering section. All TG curves almost no longer decrease, and DTG approaches 0; the system completes decomposition and begins to undergo solid-state sintering and crystal phase rearrangement, with no further change in sample quality. This transition from decomposition-controlled to diffusion-controlled behavior marks a critical milestone in the firing schedule, where further heating should focus on promoting densification and phase equilibration rather than accommodating gas release.

**Figure 7:**
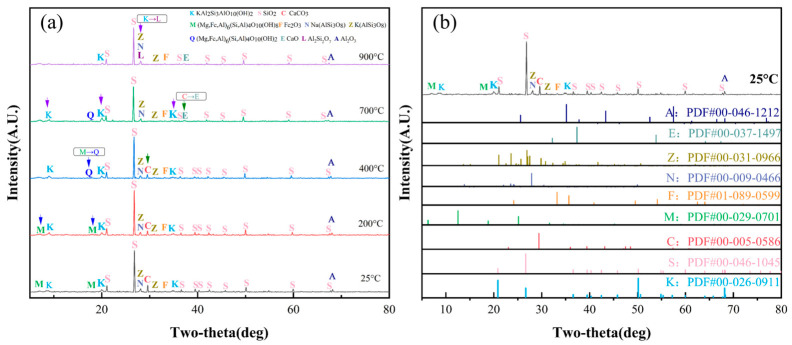
(**a**) XRD patterns of the mixture after heating at 25, 200, 400, 700, and 900 °C for 1 h, showing the evolution of mineral diffraction peaks during thermal decomposition and dehydroxylation; (**b**) the corresponding standard PDF cards for phase identification.

The physical and chemical changes occurring at three temperatures of 270.5 °C, 547.9 °C, and 767.9 °C, represented by the TG–DTG curve, need to be verified based on XRD analysis. Heat three materials in a 1:1:1 mixture at 200 °C, 400 °C, 700 °C, and 900 °C for 1 h, and observe the peak changes in XRD, as shown in Figure 7, to determine the chemical reaction in this temperature range.

From Figure 7, the following phenomenon can be observed:

(1) By comparing the XRD analysis of the mixture heated at 25 °C and 200 °C (air atmosphere) for 1 h, it was found that all phases at 25 °C still existed after heating at 200 °C, and the peak intensity of each phase increased. This indicates that the weight loss reaction expressed by thermogravimetric analysis at 119.8 °C was only the evaporation of physical water, and other molecular structures did not undergo any physical or chemical changes in the mineral phase. The increased intensity of all diffraction peaks at 200 °C is a manifestation of improved crystallinity arising from particle rearrangement rather than structural ordering. As reported by Gu et al. [16] in their study of Chinese red clay, the removal of physically adsorbed water eliminates the “dilution effect” of amorphous interparticle water, which weakens the background scattering and enhances the signal-to-noise ratio of crystalline phases. This phenomenon indicates that the thermal treatment at 200 °C is safe for DIW-printed bodies in terms of structural integrity, which is consistent with the observation by Mostafa et al. [18] that dehydroxylation does not commence below 200 °C for iron-bearing clay systems.

(2) The XRD spectra of the sample heated at 200 °C for 1 h were compared with those of the sample heated at 400 °C (air atmosphere) for 1 h. It was found that the (Mg, Fe, Al)_6_(Si, Al)_4_O_10_(OH)_8_ peak in the original material disappeared, and the (Mg, Fe, Al)_6_(Si, Al)_4_O_10_(OH)_2_ phase appeared. In other words, the (Mg, Fe, Al)_6_(Si, Al)_4_O_10_(OH)_8_ underwent a dehydroxylation reaction between 200 and 400 °C, resulting in the precipitation of three crystal waters. Combined with TG–DTG analysis, this chemical reaction occurred at 270.5 °C, as shown in Equation (1):

(1) $$(Mg,Fe,Al{)}_{6}(Si,Al{)}_{4}O_{10}(OH{)}_{8}\to (Mg,Fe,Al{)}_{6}(Si,Al{)}_{4}O_{10}(OH{)}_{2}+3H_{2}O\;↑$$

(3) After heating the material at 400 °C and continuing to heat it at 700 °C (air atmosphere) for 1 h, XRD analysis revealed that the CaCO_3_ phase disappeared from the spectrum heated at 400 °C, and a new CaO phase was added to the spectrum heated at 700 °C. Compared with TG–DTG analysis, a thermal decomposition reaction of CaCO_3_ occurred at 547.9 °C, as shown in Equation (2).

(2) $$CaCO_{3}\to CaO+CO_{2}↑$$

The indexed XRD peaks provide direct crystallographic evidence for the thermal reactions inferred from TG–DTG/DSC. In particular, the disappearance of the calcite reflection at approximately 29.3° (104) and the appearance of CaO-related reflections confirm the decomposition of CaCO_3_ into CaO and CO_2_. Reported XRD indexing of calcite assigns its main peaks to 22.9° (012), 29.3° (104), 35.9° (110), 39.3° (113), 43.0° (202), 47.4° (018), and 48.4° (116) [26]. Therefore, the weakening or disappearance of these calcite peaks in the present patterns supports the assignment of the 547.9 °C thermal event to carbonate decomposition, while the changes in clay-mineral reflections are consistent with dehydroxylation of layered silicates.

The substantial reduction observed in the present system is quantitatively explained by the particle size effect derived from the Gibbs free energy surface energy correction, as detailed in Section 3.2. The equivalent radius of CaCO_3_ in the clay matrix, calculated as 1.31 μm, yields a theoretical decomposition temperature that is in excellent agreement with the experimental value of 547.9 °C, confirming that the surface energy contribution, which scales inversely with particle radius, is the dominant factor responsible for the temperature depression.

Recent investigations provide a quantitative context for this observation. Zhuang et al. [26] established a comprehensive kinetic framework for CaCO_3_ decomposition using TG–DTG–DSC under varying heating rates and atmospheres. A combined experimental and molecular dynamics study [27] identified 1000 °C as a critical threshold for accelerated decomposition, with the Ca diffusion coefficient increasing by 3.6 times at 1473 K. Kinetic analysis under nonisothermal conditions [28] further demonstrated that the activation energy is strongly heating-rate-dependent, with calculated conversion values deviating by <4.1% from experimental data.

Crucially, the decomposition behavior of CaCO_3_ is profoundly influenced by the surrounding oxide matrix. Al_2_O_3_ nanoparticles have been shown to destabilize calcium carbonate at temperatures as low as 350 °C, forming calcium aluminate phases and releasing CO_2_ [29]. In kaolin–calcite-based ceramics, calcite decomposition is intimately coupled with solid-state reactions above 800 °C, driving significant changes in phase composition and elastic properties [30]. These findings support the interpretation that the decomposition temperature of 547.9 °C in the present clay system arises from the combined effect of particle size and the additional destabilizing influence of the Al_2_O_3_– and SiO_2_-rich matrix, which introduces interfacial strain and favorable pathways for solid-state reactions that further lower carbonate stability.

From the perspective of firing process optimization, this result has two implications. First, the decomposition temperature is not an intrinsic material constant but depends on both grinding fineness and matrix chemistry; batch-to-batch variations must be carefully controlled. Second, CO_2_ release at this reduced temperature occurs when the green body is only partially sintered, which is favorable for gas escape but still requires careful heating rate control to prevent localized accumulation—particularly critical for DIW-printed bodies with anisotropic permeability (Section 2.3).

(4) In the XRD pattern of the mixture at 900 °C, it was found that the phase strength of KAl_2_Si_3_AlO_10_(OH)_2_ decreased and the phase strength of Al_2_Si_2_O_7_ increased, as shown in Equation (3).

(3) $$KAl_{2}Si_{3}AlO_{10}{\left(OH\right)}_{2}\to 2KAl{Si}_{3}O_{8}+{Al}_{2}{Si}_{2}O_{7}+2H_{2}O$$

This indicates that the peak of the thermogravimetric experiment at 767.9 °C is the reaction of KAl_2_Si_3_AlO_10_(OH)_2_ dehydroxylation to generate Al_2_Si_2_O_7_. After undergoing dehydration at 270.5 °C to remove three crystal waters and then removing two more crystal waters at 767.9 °C, the dehydration reaction is completely completed.

### 3.2. Calculation of CaCO_3_ Decomposition Reaction Temperature in Clay

The standard Gibbs free energy for the decomposition Equation (2) of CaCO_3_ is shown in Equation (4) [23]

(4) $$\Delta G_{T}^{\emptyset }=174,923-150\;T$$

The reaction free energy in a general non-standard state is $\Delta G=\Delta G_{T}^{\emptyset }+RT\ln p_{{CO}_{2}}$. When ΔG=0, the decomposition reaction reaches equilibrium; the pressure at this point is $p_{{CO}_{2}}$. The corresponding temperature is called the decomposition temperature, as shown in Equation (5).

(5) $$T_{decompose}=\frac{174,923}{150-R\ln p_{{CO}_{2}}}$$

When the pressure $p_{{CO}_{2}}=1$ (dimensionless), the corresponding decomposition temperature is called the boiling decomposition temperature, $T_{\mathrm{decompose}}=1163\;K\;\left(890\;{}^{\circ}C\right)$, which is the starting decomposition temperature of pure calcium carbonate. At present, lime production from calcium carbonate is also carried out at this temperature, but the raw material for lime combustion is generally limestone larger than 10 mm. For very small particles, the initial decomposition temperature of CaCO_3_ will change, and this change will increase with the decrease in particle size.

In physical chemistry, the surface energy of Gibbs free energy cannot be ignored if a component is a spherical particle [31,32]. For the system consisting of K components, the Gibbs free energy is shown in Equation (6).

(6) $$G=\sum_{i=1}^{K}\left(u_{i,m}n_{i}+\sigma_{i}A_{i}\right)$$

where $u_{i,m}$ is the molar chemical potential of component i without considering the surface energy, J/mol; $\sigma_{i}$ is the surface energy of component i, J/m^2^; $A_{i}$ is the surface area per mole of component i, m^2^/mol; $n_{i}$ is the number of moles of the component. At this time, the chemical potential of i, $u_{i}$, can be expressed as Equation (7).

(7) $$u_{i}=\frac{\partial G}{\partial n_{i}}=u_{i,m}+\sigma_{i}\frac{\partial A_{i}}{\partial n_{i}}=u_{i,m}+\sigma_{i}\frac{\partial A_{i}}{\partial V_{i}}\frac{\partial V_{i}}{\partial n_{i}}$$

If the micro particle is an equivalent circle, its radius is $r_{i}$ and its volume is V, so that $\frac{\partial A_{i}}{\partial V}=\frac{\partial \left(4\pi r_{i}^{2}\right)}{\partial \left(\frac{4}{3}\pi r_{i}^{3}\right)}=\frac{2}{r_{i}}$, and then it is defined as $\frac{\partial V}{\partial n_{i}}=\bar{V_{i}}$, the partial molar volume of component i, which can also be expressed as $\bar{V_{i}}=\frac{M_{i}}{\rho_{i}}$. Assuming that $a_{i}$ is the activity of component i in the system of small particles, its reduced degree expression (7) becomes Equation (8).

(8) $$u_{i}=u_{i,m}+\frac{2M_{i}\sigma_{i}}{\rho_{i}r_{i}}=u_{i,m}^{\emptyset }+RT\ln a_{i}+\frac{2M_{i}\sigma_{i}}{\rho_{i}r_{i}}$$

Equation (8) is used to calculate the standard free energy change in the spherical CaCO_3_ decomposition reaction ${CaCO}_{3}=CaO+{CO}_{2}$ of equivalent small particles, as shown in Equation (9).

(9) $$\Delta_{r}G^{\emptyset }=u_{{CO}_{2}}^{\emptyset }+u_{CaO}^{\emptyset }-u_{{CaCO}_{3}}^{\emptyset }{=\Delta }_{r}G_{V}^{\emptyset }+(\frac{2M_{CaO}\sigma_{CaO}}{\rho_{CaO}r_{CaO}}-\frac{2M_{CaCO_{3}}\sigma_{CaCO_{3}}}{\rho_{CaCO_{3}}r_{CaCO_{3}}})$$

In the formula, $K_{V}^{\emptyset },\Delta_{r}G_{V}^{\emptyset },p_{{CO}_{2},V}$ are the equilibrium constant, standard free energy change (J), and corresponding decomposition pressure of a general large particle calcium carbonate decomposition reaction, respectively; $u_{{CO}_{2}}^{\emptyset },u_{CaO}^{\emptyset },u_{{CaCO}_{3}}^{\emptyset }$ are the standardized degrees of CO_2_ and micro particles CaO and CaCO_3_. Since the decomposition equilibrium is $\Delta G_{T}^{\emptyset }=-RT\ln p_{{CO}_{2}}$, the standard free energy Equation (4) is substituted into Equation (9), and Equation (10) is obtained.

(10) $$\ln p_{{CO}_{2}}=18.04-\frac{21,040}{T}+2(\frac{M_{{CaCO}_{3}}\sigma_{{CaCO}_{3}}}{\rho_{{CaCO}_{3}}r_{{CaCO}_{3}}}-\frac{M_{CaO}\sigma_{CaO}}{\rho_{CaO}r_{CaO}})$$

Assuming that the particle size of the tiny calcium carbonate particles is equal to the CaO obtained by decomposition, i.e., $r_{{CaCO}_{3}}=r_{CaO}$. Since it is a solid state at the time of decomposition, assuming that the molar volumes before and after decomposition are approximately equal, $\frac{M_{{CaCO}_{3}}}{\rho_{{CaCO}_{3}}}\approx \frac{M_{CaO}}{\rho_{CaO}}=\bar{V}$, the surface free energy difference before and after decomposition of small particles is $\Delta \sigma =\sigma_{{CaCO}_{3}}-\sigma_{CaO}$, and Equation (10) can be changed to Equation (11).

(11) $$\ln p_{{CO}_{2}}=18.04-\frac{21,040}{T}+\frac{{\bar{V}}_{{CaCO}_{3}}}{r_{{CaCO}_{3}}}\Delta \sigma$$

Equation (11) is the central result of this thermodynamic derivation. It reveals that the decomposition pressure of micron-sized CaCO_3_ is elevated by the term $\frac{{\bar{V}}_{{CaCO}_{3}}}{r_{{CaCO}_{3}}}\Delta \sigma$, relative to the bulk material. Since $\Delta \sigma =\sigma {CaCO}_{3}-\sigma CaO>0$ (the surface energy of CaCO_3_ is higher than that of CaO), the logarithmic decomposition pressure increases as the particle radius decreases. At a fixed external CO_2_ pressure ($p_{{CO}_{2}}=1$), this increase in decomposition pressure necessarily results in a lower decomposition temperature. Physically, the high surface energy of fine CaCO_3_ particles makes the solid phase less stable thermodynamically, so decomposition to CaO becomes favorable at a lower temperature. This is the thermodynamic origin of the particle size effect: the surface energy penalty of the reactant ($\sigma {CaCO}_{3}$) exceeds that of the product (σCaO), and this energy difference, amplified by the 1/r scaling of the surface-to-volume ratio, drives the decomposition temperature downward as the particle size decreases [33]. This theoretical framework has been validated experimentally for various nanomaterials, where the onset decomposition temperature has been shown to decrease linearly with the reciprocal of particle size [34,35].

It can be seen that the decomposition pressure of microparticle calcium carbonate increases by $\frac{{\bar{V}}_{{CaCO}_{3}}}{r_{{CaCO}_{3}}}\Delta \sigma$, the decomposition pressure increases, and the decomposition temperature decreases. According to the difference between the surface energy before and after the decomposition of calcium carbonate, given in the document [23], $\Delta \sigma =0.50\;J/m^{2},{\bar{V}}_{CaCO_{3}}=2\times 10^{-5}\;m^{3}/mol$, and the decomposition temperature of CaCO_3_ with different particle sizes $r_{{CaCO}_{3}}$ at $P_{{CO}_{2}}=1$ can be calculated, as shown in Table 2.

It can be seen from Table 2 that when the radius of CaCO_3_ is 1 μm, the decomposition temperature can be reduced from 890 °C to 477 °C, while the actually measured CaCO_3_ in clay decomposes at 547.9 °C. It can be inferred that the radius of CaCO_3_ in clay is between 1 and 10 μm, and the corresponding equivalent radius of calcium carbonate calculated from Equation (11) is 1.31 μm.

With thermal and phase analyses as the core means, the physicochemical changes and key reaction mechanism of the Hejin gray pottery ternary mixture during the heating process were studied, which is also a form of kiln change. The combined analyses of TG–DTG, DSC and XRD confirmed that the four temperature points of the mixed green body were 119.8 °C (volatilization of free water), 270.5 °C (precipitation of three crystal waters), 547.9 °C (decomposition of CaCO_3_ to produce CaO and release CO_2_), and 767.9 °C (precipitation of two crystal waters, complete dehydroxylation and generation of the metakaolinite phase). These four temperature points were the key nodes leading to the cracking, foaming, and deformation of the green body during the firing process in the kiln. In this chapter, the thermal stability of raw materials and the causes of defects are clarified from the perspective of molecular structure evolution, which provides a direct theoretical basis for the subsequent development of a reasonable temperature rise curve, the suppression of firing defects, and the optimization of reduction atmosphere and temperature regime.

## 4. Mechanism Analysis of Material Color Change in Kiln During the Firing Process of Gray Pottery

The color of the top six oxides with the most content in the three raw materials used for gray pottery products at room temperature is shown in Table 3. It can be seen that except for iron oxide, which is reddish brown, other pure SiO_2_, K_2_O, MgO, CaO, and Al_2_O_3_ are transparent or white. The content of Fe_2_O_3_ in the three raw materials of Hejin gray pottery is, respectively, 44.2% of purple sand, 6.90% of steel mud, and 8.19% of clay. According to the ratio of 1:1:1, the mixture contains 19.8% of Fe_2_O_3_, which is the reason for its red color. However, the most representative color of the samples fired in the kiln is dark gray or iron blue. As such, how is the color controlled in the firing process and how does it change from gray to iron blue as required?

**Figure 8:**
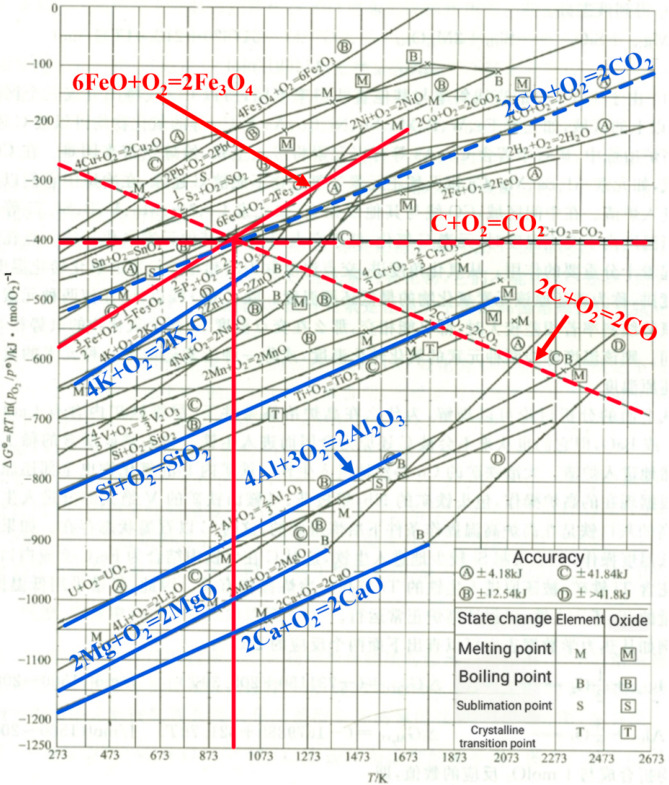
Ellingham diagram.

As an intangible cultural heritage, Hejin gray pottery is traditionally fired with coal or wood used as fuel. Both of them have dual functions of being heating and reducing agents in the high-temperature environment of a kiln. The reducing atmosphere in the kiln mainly comes from CO and H_2_ generated by incomplete combustion of coal or wood. Taking the oxidation process of the main element carbon (C) in coal as an example, carbon reacts with oxygen (O_2_) in the air to generate both CO_2_ and CO; the generation of CO corresponds to a reducing atmosphere, while the generation of CO_2_ corresponds to an oxidizing atmosphere. The thermodynamic trend of the reaction can be analyzed by the Ellingham diagram (Figure 8) [36].

### 4.1. Qualitative Analysis of the Color Change of Gray Pottery Raw Materials in the Kiln When C Is the Fuel

In 1944, Ellingham used the standard Gibbs free energy of formation of oxides $\Delta_{f}G_{M_{x}O_{y}}^{\emptyset }$ to calculate the standard Gibbs free energy $\Delta_{r}G_{E}^{\emptyset }$ of the reaction of common elemental element M in nature with 1mol oxygen as a function of temperature, as shown in Equation (12).

(12) $$\frac{2x}{y}M+O_{2}=\frac{2}{y}M_{x}O_{y}\;\Delta_{r}G_{M_{x}O_{y}}^{\emptyset }=a+bT$$

The relationship between the standard Gibbs free energy change in these reactions and temperature is plotted as an Ellingham diagram, also known as an oxygen potential diagram. Three conclusions can be drawn from the diagram: at the same temperature, when some elementary substances react with 1 mol O_2_, the lower the position in the diagram, the stronger the oxidation trend of the elementary substances, and the more favorable the reaction. At a certain temperature, the lower element can reduce the oxide of the higher element; the relative positions of most reaction lines do not change with temperature, and only a few reactions (such as oxidation of carbon) will cross and change the relative order with the increase in temperature. Although the above conclusion is a qualitative judgment, it accurately reflects the thermodynamic trend of the oxidation-reduction reaction between substances [36].

When Hejin gray pottery raw material is fired with coal, the changes in the kiln can be analyzed by using the Ellingham diagram:

(1) The reaction conditions of C and O_2_ to form CO and CO_2_ can be judged. On the Ellingham diagram, two red dashed lines are the curves of the standard Gibbs free energy $\Delta_{r}G_{E}^{\emptyset }$ of CO and CO_2_ generated by the reaction of C with O_2_ as a function of temperature. They have an intersection at 973 K (700 °C). When the temperature in the kiln is less than 700 °C, C reacts with O_2_ to generate CO_2_; when the temperature in the kiln is greater than 700 °C, C reacts with O_2_ to generate CO; when the temperature in the kiln is equal to 700 °C, CO and CO_2_ are generated at the same time. Therefore, the temperature in the kiln needs to be greater than 700 °C in order to produce a reducing CO atmosphere in the reaction of C and O_2_.

(2) When the temperature in the kiln is above 700 °C, the reduction ability of CO generated by coal combustion to oxides can be analyzed by the Ellingham diagram. According to the basic principle of the Ellingham diagram, the lower the curve position of the standard Gibbs free energy change $\Delta_{r}G_{E}^{\emptyset }$ with temperature, the stronger the thermodynamic trend of the corresponding oxidation reaction, so the reductant with a lower curve position can reduce the oxide with a higher position. Comparing the $\Delta_{r}G_{E}^{\emptyset }$ curve of CO oxidation to CO_2_ with temperature (blue dotted line in the figure), with the oxidation reaction curves of Fe (red solid line) and K, Si, Al, Mg, and Ca (blue solid line), it can be seen that only the oxidation reaction curves of Fe and CO intersect at 700 °C. When the temperature in the kiln is higher than 700 °C, the $\Delta_{r}G_{E}^{\emptyset }$ of CO oxidation to CO_2_ is lower than that of the Fe oxidation reaction. At this time, CO has the thermodynamic conditions for reducing Fe_2_O_3_. The oxidation reaction curves of K, Si, Al, Mg, Ca, and other elements are always below the CO curve, and their oxides cannot be reduced by CO.

(3) When the temperature is higher than 700 °C, the line of CO_2_ generated by the reaction of CO and O_2_ is much higher than that of K, Si, Al, Mg, Ca, and O_2_, so CO cannot reduce K_2_O, SiO_2_, Al_2_O_3_, MgO, and CaO in the raw materials of gray pottery, but can only reduce Fe_2_O_3_, as rationalized by the thermodynamic framework of the Ellingham diagram.

Generally, there are three forms of Fe oxides. The highest price is Fe_2_O_3_ (red), followed by Fe_3_O_4_ (gray), and the lowest price is FeO (black). It can be qualitatively considered that if coal is used as raw material during the firing of gray pottery, the temperature in the kiln is greater than 700 °C, which is the necessary condition for the color change in the kiln. In addition to iron oxides, K, Si, Al, Mg, Ca, and other oxides cannot be reduced by CO. The color of gray pottery products is only related to the valence state of iron.

Due to the uneven temperature in the kiln, CO_2_ will be generated at places below 700 °C, which may lead to a mixed atmosphere of CO + CO_2_ in the kiln. Therefore, it is necessary to accurately calculate the mechanism of raw material kiln change under the mixed atmosphere of CO + CO_2_.

### 4.2. Quantitative Analysis of the Color Change of Gray Pottery Raw Materials in the Kiln When C Is the Fuel

The thermodynamics research on the reduction of iron oxide in iron ore by CO has been very mature, but the research on the reduction of iron oxide with a lower concentration in too-gray pottery is less. Pure iron oxide has the “step-by-step reduction principle”, and the step-by-step reduction of iron oxide by CO from the highest price to the lowest price is shown in Equations (13)–(15) [36].

(13) $$3{Fe}_{2}O_{3}+CO=2{Fe}_{3}O_{4}+{CO}_{2}\;\Delta_{r}G_{13}^{\emptyset }=-26,520-57.03\;T$$

(14) $${Fe}_{3}O_{4}+CO=3FeO+{CO}_{2}\;\Delta_{r}G_{14}^{\emptyset }=35,120-41.53\;T$$

(15) $$FeO+CO=Fe+CO_{2}\;\Delta_{r}G_{15}^{\emptyset }=-17,500+21.00\;T$$

Equations (13)–(15) reveal that Fe_3_O_4_ → FeO (reaction 14) has ΔG° = 35,120 − 41.53 T, becoming negative only above ~846 K (573 °C)—explaining why FeO is unstable below ~570 °C. Reaction (15), FeO → Fe, has ΔG° = −17,500 + 21.00 T, positive above ~560 °C, making metallic iron formation thermodynamically unfavorable at typical firing temperatures. These thermodynamic constraints on iron oxide reduction in CO-containing atmospheres have been confirmed by recent kinetic studies [37].

Because multivalent oxides have the “principle of existence of the lowest oxide”, the lowest oxide of iron is FeO, and the lowest temperature of its existence is 570 °C. When the temperature is lower than 570 °C, FeO cannot exist, and Fe_3_O_4_ directly passes through FeO to reduce to Fe, and the reaction is Equation (16).

(16) $$\frac{1}{4}{Fe}_{3}O_{4}+CO=\frac{3}{4}Fe+{CO}_{2}\;\Delta_{r}G_{16}^{\emptyset }=0.17\;T$$

When coal is used as fuel, the temperature in the kiln must be controlled above 700 °C to produce a CO-reducing atmosphere. Although the firing of Hejin gray pottery does not need a temperature of less than 570 °C, due to the uneven temperature distribution of the kiln firing gray pottery, CO_2_ will be generated in the area where the temperature is less than 700 °C, so it is necessary to calculate the conditions for the first-order reaction of Fe_2_O_3_ (13) CO and CO_2_ partial pressure by thermodynamics.

The free energy of reaction (13) in a non-standard state is shown in Equation (17).

(17) $$\Delta_{r}G_{13}=\Delta_{r}G_{13}^{\emptyset }+RT\ln\frac{p_{CO}}{p_{CO}+p_{{CO}_{2}}}$$

Among them, $p_{CO}$ and $p_{{CO}_{2}}$ are dimensionless partial pressures of CO and CO_2_ in the gas phase, respectively.

When the reaction reaches equilibrium, $\Delta_{r}G_{13}^{\emptyset }=-RT\ln\frac{p_{CO}}{p_{CO}+p_{{CO}_{2}}}$, can be obtained. By using the logarithmic transformation relationship and substituting into Equation (17), Equation (18) is obtained:

(18) $$-26,520-57.03T=-RT\ln\frac{p_{{CO}_{2}}}{p_{CO}}$$

The linear relationship between equilibrium partial pressure ratio and temperature can be obtained by further sorting Equation (18), namely Equation (19):

(19) $$\ln\frac{p_{CO_{2}}}{p_{CO}}=\frac{3190}{T}+6.86$$

If the temperature is 800 °C (1073 K) and 900 °C (1173 K), $\frac{p_{CO_{2}}}{p_{CO}}$ in the gas phase is 18,583 and 14,472, respectively, calculated from Equation (19).

Assuming $p_{CO}+p_{CO_{2}}=1$, the partial pressure $\frac{p_{CO}}{p_{CO}+p_{{CO}_{2}}}$ of CO in the equilibrium atmosphere at 800 °C and 900 °C is 5.38 × 10^−5^ and 6.91 × 10^−5^, respectively. In other words, Fe_2_O_3_ can be reduced to Fe_3_O_4_ by a very small amount of CO partial pressure in the gas phase. If the temperature is taken as the abscissa and the CO partial pressure is plotted as the ordinate, this line almost coincides with the abscissa on the graph.

**Figure 9:**
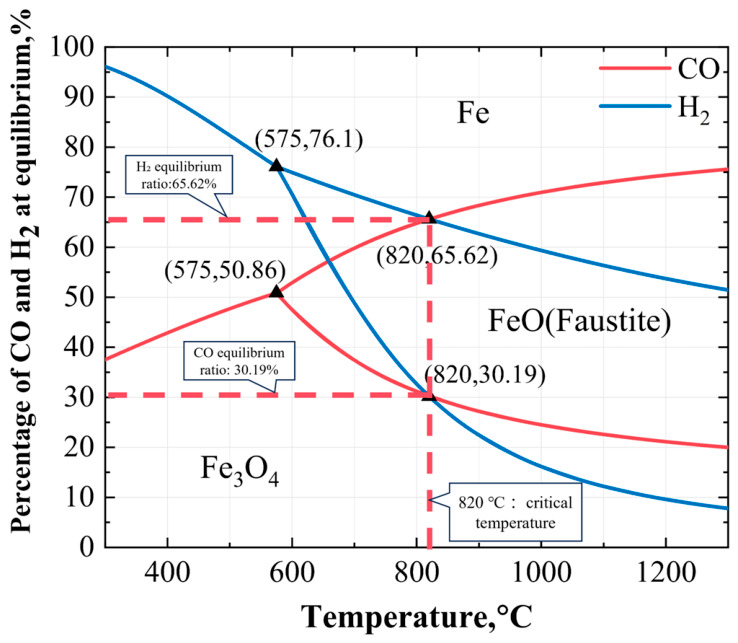
Equilibrium diagram of CO and H_2_ reduction of iron oxides.

Similarly, the values of the relationship between $\frac{p_{CO}}{p_{CO}+p_{{CO}_{2}}}$ and T, calculated by Equation (16) at a temperature less than 570 °C, and Equations (14) and (15) at a temperature greater than 570 °C, are drawn in the same figure, as shown in Figure 9, which is the famous “fork curve (red)”. In the same way, the second “fork curve (blue)” in Figure 9 can be obtained by replacing CO with H_2_ as a reductant [36].

When firing Hejin gray pottery, the color change of gray pottery from gray to black can be obtained by controlling the atmosphere in the kiln according to the “fork curve”, in theory. If coal is used as fuel and the temperature in the kiln is 900 °C, gray products are expected. The reduction of Fe_2_O_3_ to Fe_3_O_4_ is controlled, and $\frac{p_{CO}}{p_{CO}+p_{{CO}_{2}}}$ in the atmosphere in the kiln should not be higher than 0.28. If gray pottery needs to be darker, $\frac{p_{CO}}{p_{CO}+p_{{CO}_{2}}}$ should be controlled so as to be greater than 0.28 and less than 0.68. At this time, the oxide of iron is FeO. If $\frac{p_{CO}}{p_{CO}+p_{{CO}_{2}}}$ is greater than 0.68, iron particles will be produced in gray pottery products. This is the ideal condition for a reduction of pure iron oxide by CO; however, the actual situation still needs experimental verification.

The “fork curve” in Figure 9 describes the equilibrium conditions for pure iron oxide reduction. In the Hejin system, however, the presence of ~12.82 wt% Al_2_O_3_ intimately mixed within the clay matrix fundamentally alters this pathway. FeO generated by initial reduction preferentially reacts with Al_2_O_3_ to form FeAl_2_O_4_ spinel rather than proceeding along the stepwise Fe_3_O_4_ → FeO → Fe sequence. This competitive reaction “traps” Fe^2+^ in a thermodynamically stable spinel, preventing further reduction. The FeAl_2_O_4_ remains stable across 20–90% CO, which is consistent with recent findings on iron-enriched kaolin under oxygen-deficient atmospheres [38].

### 4.3. Experimental Verification of Material Color Change in 3D Printing Gray Pottery Kiln

The raw materials of Hejin gray pottery were ground to 200 mesh and then added to 24–27% water for 3D printing. The printing speed was 40 mm/s, the layer thickness was 0.7 mm, and the filling rate was 70%. The samples were printed into three models of (60 × 10 × 4) mm, (10 × 10 × 10) mm, and Φ 10 × 12.5. After drying, the samples were fired in electric kilns with different atmospheres. The kiln-transformation mechanism of gray pottery under different firing conditions was studied to provide a theoretical basis for the optimal firing process.

**Figure 10:**
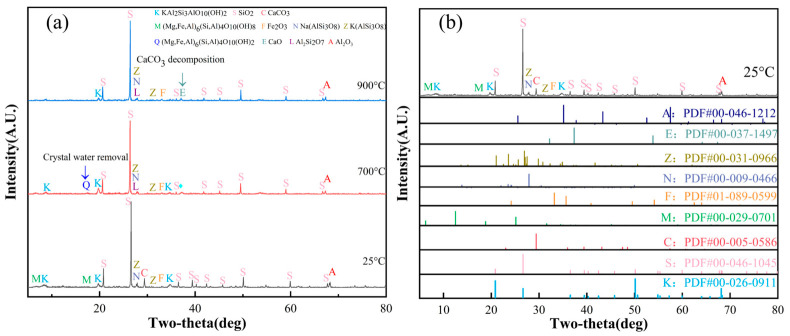
XRD patterns of the mixed material at 25 °C, 700 °C, and 900 °C (**a**), and the corresponding standard PDF reference patterns (**b**).

According to the “fork curve” of the oxides of CO-reduced iron and the Ellingham diagram in Figure 8, the experimental scheme of gray pottery kiln transformation is formulated. For comparison, firstly, the lime ceramic mixture (steel mud: clay: purple sand = 1:1:1) with a particle size of 100 mesh was placed in a tubular furnace (air atmosphere) and fired at 700 °C and 900 °C for 1 h, respectively. XRD analysis was used to detect the phase composition of the mixture at room temperature and after firing. As shown in Figure 10, it was found that Fe_2_O_3_ was contained in the mixture and was stable at three temperatures. Under a fully oxidizing air atmosphere, no diffraction signals of any divalent iron-bearing minerals can be detected, and Fe_2_O_3_ remains the only iron-containing phase. This directly proves that a reducing environment above 700 °C is an indispensable prerequisite for forming the characteristic celadon-gray phase of Hejin gray pottery.

**Figure 11:**
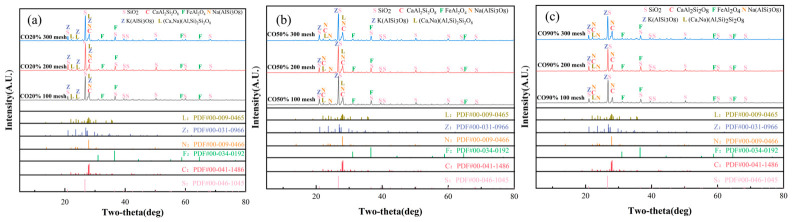
The phase composition of the samples under different CO concentration firing atmospheres at (**a**) 20%, (**b**) 50%, and (**c**) 90%. Only diffraction peaks assigned to FeAl_2_O_4_ spinel are observed in all three groups.

Grind the above mixture to 100 mesh, 200 mesh, and 300 mesh sizes, and place it in a tubular furnace with 20%, 60%, and 90% CO concentration, respectively, and control the temperature at 900 °C for one hour. XRD analysis was carried out on the reduced fired samples to detect whether the molecular structure generated Fe_3_O_4_, FeO, or Fe. The experimental results are shown in Figure 11.

The dominant diffraction peaks in Figure 11 were indexed to hercynite FeAl_2_O_4_ with a spinel structure, corresponding mainly to the (220), (311), (400), (422), (511), and (440) crystal planes. No obvious reflections corresponding to magnetite, wüstite, or metallic Fe were detected. This result differs from the classical reduction sequence of pure iron oxide, Fe_2_O_3_ → Fe_3_O_4_ → FeO → Fe, reported for Fe_2_O_3_ reduction in H_2_–CO atmospheres [37]. The preferential formation of FeAl_2_O_4_ indicates that, in the Al_2_O_3_-rich Hejin gray pottery matrix, reduced Fe^2+^ is stabilized as hercynite spinel rather than continuing along the pure iron-oxide reduction pathway. This phase assignment is also consistent with reported high-temperature XRD studies of FeAl_2_O_4_ spinel [39].

From the experimental results in Figure 11, an abnormal phenomenon is found. For the sample fired under 20% CO, Fe_2_O_3_ should be reduced to Fe_3_O_4_ [40]; however, the only iron that contains oxide in the sample is FeAl_2_O_4_. For the samples sintered at 50% CO, Fe_2_O_3_ should be reduced to FeO, and the only oxide that contains iron in the samples is FeAl_2_O_4_. For the samples sintered at 90% CO, Fe_2_O_3_ should be reduced to metal Fe, but the only oxide that contains iron in the samples is FeAl_2_O_4_. Why is this?

Consistent with the phase evolution rule of iron-rich aluminosilicate clay under low-oxygen sintering conditions reported by Guillemin et al. [38], Fe^3+^ ions reduced to Fe^2+^ tend to preferentially form hercynite (FeAl_2_O_4_) spinel in high-alumina clay substrates, which inhibits the classical sequential reduction path of pure iron oxides to generate magnetite or wüstite. Even when the partial pressure of CO varies over a wide range, hercynite always acts as the dominant iron-containing crystal phase, which is highly consistent with the XRD test results of samples fired under 20–90 vol% CO in this work.

According to the Gibbs free energy data listed in Table 4, the reaction between FeO and Al_2_O_3_ to generate FeAl_2_O_4_ releases far more free energy than the reaction between FeO and residual Fe_2_O_3_ to form Fe_3_O_4_. As long as CO reduces Fe_2_O_3_ to produce FeO, FeO will preferentially combine with abundant Al_2_O_3_ distributed in the matrix instead of forming the magnetite intermediate phase. This competitive solid-state reaction thoroughly breaks the classical stepwise Fe_2_O_3_ → Fe_3_O_4_ → FeO reduction law summarized from pure iron oxide systems.

Moreover, XRD patterns of samples treated with 20–90 vol% CO show no obvious difference in the position and intensity of FeAl_2_O_4_ diffraction peaks. It can be judged that the Fe-Al spinel phase maintains high thermodynamic stability within this wide CO concentration range, and the final gray tone barely changes with CO content.

Even under a 90 vol% high-concentration CO atmosphere, no diffraction peaks of metallic iron can be captured on the XRD spectrum. As reflected by Equation (22), reducing FeAl_2_O_4_ into elemental iron requires an extremely high temperature far beyond 900 °C, so the compact spinel crystal structure cannot be destroyed by CO under our sintering conditions.

According to the “fork curve” of the reduction reaction between pure Fe_2_O_3_ and CO, Fe_3_O_4_ should be produced in the sample when the concentration of CO is 20%, but it can be seen from reaction (20) that Fe_3_O_4_ is actually produced by the reaction between FeO and Fe_2_O_3_. It can be seen from Table 4 that there is 12.92% Al_2_O_3_ in the mixture. From the calculation of the standard Gibbs free energy of Equations (20) and (21) at all temperatures between 973 K and 1273 K, it is found that the negative value of Gibbs free energy of the combination of Al_2_O_3_ and FeO is much greater than that of Fe_2_O_3_ and FeO. Therefore, Fe_3_O_4_ is not formed in the samples sintered at the concentration of 20% CO, and FeAl_2_O_4_ is replaced.

(20) $$FeO+Fe_{2}O_{3}=Fe_{3}O_{4}\;\Delta G^{\emptyset }=-22,200+1.75\;T$$

(21) $$\mathrm{FeO}+{\mathrm{Al}}_{2}O_{3}=\mathrm{FeO}\cdot {\mathrm{Al}}_{2}O_{3}\;\Delta G^{\emptyset }=-59,204+22.343\;T$$

The data in Table 4 quantitatively explain the preferential FeAl_2_O_4_ formation. At all temperatures (973–1273 K), ΔG° for FeO–Al_2_O_3_ (Equation (21)) is 10–17 kJ/mol more negative than for FeO–Fe_2_O_3_ (Equation (20)). This energy advantage means FeO, once generated, is immediately “trapped” by Al_2_O_3_ to form FeAl_2_O_4_ rather than aggregating into Fe_3_O_4_. This thermodynamic preference is consistent with the CALPHAD-based thermodynamic framework recently established for mixed oxide systems, which demonstrated that the chemical activity of oxide components can be significantly altered through mixing with other oxides, thereby modifying the thermodynamic conditions for reduction [41].

For the samples fired at 900 °C, when the concentration of CO is 60%, it can be seen from Table 3 that the negative Gibbs free energy of FeO obtained by the reduction reaction of Fe_2_O_3_ with CO and FeAl_2_O_4_ produced by the reaction of Fe_2_O_3_ with Al_2_O_3_ in the mixture is very large, which naturally disappears and is replaced by FeAl_2_O_4_.

For the sample sintered at 900 °C, when the concentration of CO is 90%, iron should be obtained by reduction according to the fork curve. Why is the test result of XRD for the reduced sample still FeAl_2_O_4_, but no Fe is found? According to the step-by-step reduction rule of oxides, when iron oxides are reduced from Fe_2_O_3_ to Fe_3_O_4_, and then to FeO, due to the presence of Al_2_O_3_, FeAl_2_O_4_ will continue to form at the stage of reduction to Fe_3_O_4_ and after. From reaction (22), it can be seen that for the standard free energy to become negative, temperature T needs to be greater than 6530 K. Therefore, while pure Fe_2_O_3_ reduces in a high-temperature reducing CO atmosphere, in the presence of Al_2_O_3_, when the CO concentration increases from 20% to 90%, the red Fe_2_O_3_ in all samples sintered at 900 °C transforms into a characteristic black FeAl_2_O_4_ spinel, but does not change into either gray Fe_3_O_4_ or black FeO.

(22) $${FeAl}_{2}O_{4}+CO=Fe+{Al}_{2}O_{3}+{CO}_{2}$$

In this case, the reason for the color change in the firing kiln “Fe_2_O_3_ (red) → Fe_3_O_4_ (ash) → FeO (black)” becomes “Fe_2_O_3_ (red) → FeAl_2_O_4_ (black)” (which is not affected by the concentration of CO in the gas).

The color of gray pottery can only be controlled by the content of Fe_2_O_3_ in the mixture. The higher the content of Fe_2_O_3_, the darker the color of gray pottery, and vice versa.

It must be pointed out that after the color change in the materials in the gray pottery kiln is completed, a necessary prerequisite to maintain the color is to cool the gray pottery under isolated air conditions after firing, so as to fix the color formed. If the firing is completed and the burnt product is placed in the atmosphere, Fe_3_O_4_ or FeO will be oxidized to Fe_2_O_3_ again, and the gray will turn red. If sintered samples are exposed to air when cooling below 600 °C, Fe^2+^ inside FeAl_2_O_4_ will be re-oxidized into Fe^3+^, and characteristic Fe_2_O_3_ diffraction peaks will reappear on XRD patterns, accompanied by an undesirable reddish surface color. This re-oxidation is thermodynamically driven: 4FeAl_2_O_4_ + O_2_ → 2Fe_2_O_3_ + 4Al_2_O_3_ becomes favorable when exposed to air (pO_2_ ≈ 0.21 atm) above ~600 °C. The inherent instability of FeAl_2_O_4_ in oxidizing atmospheres at elevated temperatures has been demonstrated by Jastrzębska et al. [39], who reported that pure hercynite (FeAl_2_O_4_) begins to decompose in air at 700 °C, with hematite (α-Fe_2_O_3_) and alumina (Al_2_O_3_) appearing as the decomposition products. Furthermore, Guillemin et al. [6] observed that in iron-enriched kaolin systems, an oxygen-deficient atmosphere leads to the formation of hercynite (FeAl_2_O_4_), in contrast to the hematite (Fe_2_O_3_) observed under oxidizing conditions. These findings collectively establish that FeAl_2_O_4_ must be protected from air exposure during cooling to prevent its re-oxidation to Fe_2_O_3_ and also define 600 °C as the critical threshold below which air isolation is required to preserve the characteristic celadon-gray color. Cooling under isolated air is mandatory to stabilize the FeAl_2_O_4_ crystalline phase and fix the uniform gray appearance.

In this chapter, the essence of kiln color change and the color control mechanism of Hejin gray pottery in reducing atmosphere are systematically expounded. Based on thermodynamic analysis, iron oxide reduction balance, and experimental verification, the core mechanism and control conditions of the formation of blue gray are defined. The research shows that the color of Hejin gray pottery comes from the valence change of Fe_2_O_3_ in the raw material. Under the condition of a CO-reducing atmosphere and >700 °C, the valence of Fe_2_O_3_ is regulated according to the step-by-step reduction law, showing a color change from red to gray and then to black.

Affected by the high content of Al_2_O_3_ in the body of the green body, the reduction product is not traditional Fe_3_O_4_ and FeO; however, it preferentially generates the FeAl_2_O_4_ spinel phase with more stable thermodynamics so that the gray pottery finally presents a uniform cyan gray, and the color tone is basically unaffected by the CO concentration (20–90%), which is only determined by the original Fe_2_O_3_ content. At the same time, it is confirmed that the color change in the kiln must be coordinated with high-temperature isolated air cooling to fix the color and prevent secondary oxidation and reddening.

From the point of view of thermodynamics, reaction kinetics, and phase competition, the internal correlation of “atmosphere temperature valence color” of Hejin gray pottery is fully revealed, which provides a core theoretical support for stabilizing hair color, eliminating color differences, and realizing color standardization.

## 5. Conclusions

This work takes Hejin gray pottery, a national intangible cultural heritage with highly empirical traditional firing technology, as the research object. Aiming at three core bottlenecks, including inconsistent raw material batches, low precision of conventional hand-forming, and ambiguous coupled kiln-transformation mechanisms, DIW additive manufacturing combined with atmosphere-controlled electric kiln sintering was adopted. TG–DTG/DSC, XRF, and XRD characterization, based on diffraction peak shifts, relative peak intensities, and full width at half maximum (FWHM), together with thermodynamic Gibbs free energy calculations, were employed to reveal the interrelated defect evolution and Fe-bearing phase competitive color-forming mechanisms of ternary local raw material printed bodies [26,30]. The major findings and contributions are summarized as follows:

(1) Combined thermal analysis and variable-temperature XRD results identified four critical thermal nodes during heating: free water evaporation at 119.8 °C, two-stage layered silicate dehydroxylation at 270.5 °C and 767.9 °C, and low-temperature CaCO_3_ decomposition at 547.9 °C. The micron-sized calcite (equivalent radius = 1.31 μm) with high surface energy reduces its equilibrium decomposition temperature from 890 °C to 547.9 °C, which is quantitatively interpreted by surface-corrected CaCO_3_ decomposition thermodynamics [30]. Distinct from isotropic hand-molded blanks, the layered anisotropic architecture of DIW-printed bodies creates tortuous, weak interlayer gas channels. Continuous H_2_O and CO_2_ released at the four characteristic temperatures cannot diffuse out smoothly, leading to unique interlayer cracking and blistering defects rarely observed in traditional pottery. The four temperature thresholds provide clear optimization targets for staged heating schedules to suppress sintering deformation.

(2) Above 700 °C, CO can only reduce Fe_2_O_3_ rather than K_2_O, SiO_2_, Al_2_O_3_, MgO, or CaO according to the Ellingham diagram thermodynamic rules [36]. Different from the classical sequential Fe_2_O_3_ → Fe_3_O_4_ → FeO reduction law summarized from pure iron oxide systems, sufficient Al_2_O_3_ in the ternary clay matrix drives Fe^2+^ (reduced from Fe_2_O_3_) to preferentially form thermodynamically stable hercynite FeAl_2_O_4_ via favorable solid-state reactions, thoroughly suppressing magnetite and wüstite generation [25]. The XRD spectra of samples sintered under 20–90 vol% CO all feature exclusive FeAl_2_O_4_ diffraction signals, with negligible variations in spinel peak position and FWHM, indicating that the CO concentration barely affects spinel crystallinity nor the final celadon-gray hue. Thermodynamic calculations prove that FeAl_2_O_4_ can only decompose into metallic iron at an ultrahigh temperature (>6530 K); therefore, such high-concentration CO cannot produce black iron particles under 900 °C firing. In addition, rapid secondary oxidation of Fe^2+^ occurs below 600 °C when exposed to air, so air-isolated cooling is mandatory to avoid reddening defects originating from re-formed hematite [39].

(3) The coupled logical chain of low-temperature mineral decomposition → DIW layered pore channel evolution → high-temperature competitive spinel formation fully clarifies the internal correlation between firing schedule, atmosphere condition, microstructure, and final color. This study breaks the empirical production mode of Hejin gray pottery and builds a full digital control system process covering raw material proportioning, DIW printing parameters, staged heating curves, and reducing atmosphere regulation. It delivers systematic theoretical guidance for standardized mass production of architectural brick carvings, roof ornaments, and cultural creative heritage ceramics, and promotes the modern industrial inheritance of this national intangible cultural heritage.

This research only investigates a fixed 1:1:1 ternary raw material ratio and a single 0.7 mm printing layer thickness. Subsequent work can expand variable raw material blending ratios and multi-scale DIW layer heights to establish a wider composition–process–property database. In addition, all atmosphere experiments were completed in atmosphere-controlled electric kilns, and the fluctuation of internal atmosphere in actual industrial kilns was not simulated; follow-up research can carry out matching tests on industrial kilns to further optimize process parame.ters. Moreover, CIELab* color quantitative characterization and mechanical strength testing can be supplemented to enrich the performance evaluation system of printed gray pottery products.

## Figures and Tables

**Table 1:** Chemical composition of the three main raw materials used for gray pottery.

	Purple Sand/%	Clay/%	Steel Mud/%	1:1:1 Mixture
Fe_2_O_3_	44.2	6.90	8.19	19.76
SiO_2_	26.35	53.19	64.435	47.99
K_2_O	5.09	2.63	4.00	3.91
Al_2_O_3_	4.99	13.50	19.98	12.82
MgO	0.97	2.01	0.99	1.32
CaO	0.86	19.00	0.42	6.76
TiO_2_	0.59	1.24	1.26	1.03
Na_2_O	0.097	1.01	0.53	0.55
P_2_O_5_	0.061	0.18	0.035	0.09
PbO	0.036			0.01
SrO	0.023	0.046	0.014	0.03
ZrO_2_	0.015	0.053	0.04	0.04

**Table 2:** Decomposition temperatures corresponding to different CaCO_3_ particle sizes.

R/m	T/k	T/°C
10^−4^	1159	886
10^−5^	1105	832
10^−6^	750	477
10^−7^	178	−95

**Table 3:** Color at room temperature of the top six oxides in terms of content in the raw materials.

Chemical Formula	Name	Normal Temperature Color	Description of Properties and Impurities
Fe_2_O_3_	Iron oxide	Reddish brown	The main color-developing oxides can show a red hue with a change in crystal form and impurities.
SiO_2_	Silicon dioxide	Water clear	The pure state is colorless and transparent; the powder is white, and impurities can cause grayish-white and light-yellow tones.
K_2_O	Potassium oxide	White	The pure product is white; light color deviation is caused by trace impurities, easily deliquescent, and rarely exists alone.
Al_2_O_3_	Alumina	White	The matrix is white, and different ions can present red and blue characteristic colors.
MgO	Magnesium oxide	White	Pure white matrix; trace impurities can cause gray and yellowish color deviation.
CaO	Calcium oxide	White	Pure white, with iron impurities; it will show light yellow and light gray tones.

**Table 4:** Gibbs free energy of FeO reacting with Fe_2_O_3_ and Al_2_O_3_ at different temperatures.

T	$\Delta G_{20}^{\emptyset }$	$\Delta G_{21}^{\emptyset }$
973	−20,497	−37,464
1073	−20,322	−35,230
1173	−20,147	−32,996
1273	−19,972	−30,761

## Data Availability

The original contributions presented in this study are included in the article. Further inquiries can be directed to the corresponding author.
